# Supplementary material for: Causal Relationship between Aging and Anorexia Nervosa: A White-Matter-Microstructure-Mediated Mendelian Randomization Analysis
Source: Biomedicines. 2024 Aug 16;12(8):1874. doi: 10.3390/biomedicines12081874 (PMC11351342; doi:10.3390/biomedicines12081874)
Supplement: Supplementary file 1 [file biomedicines-12-01874-s001.zip › Supplementary Figure/Figure S4/Singe SNP plot of MR analysis for TL to ubm-b-1853.pdf]

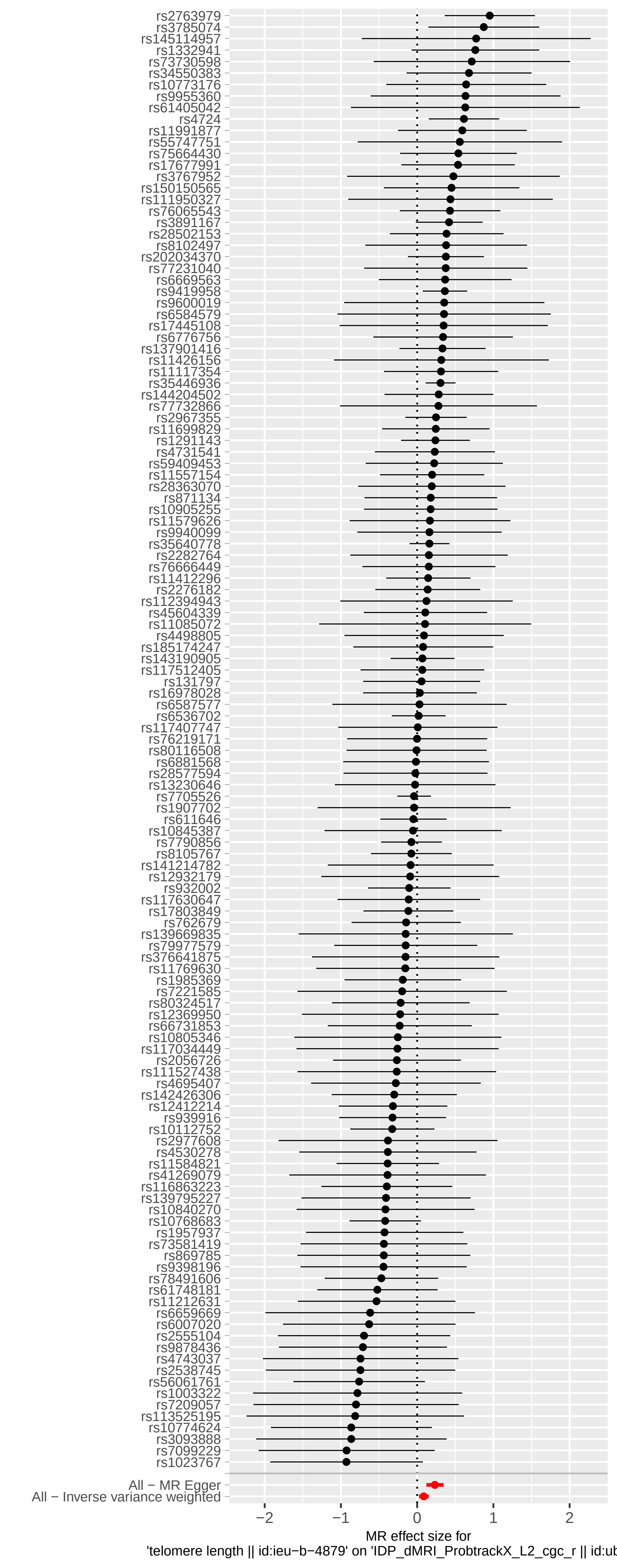
rs2763979
rs3785074
rs145114957
rs1332941
rs73730598
rs34550383
rs10773176
rs9955360
rs61405042
rs4724
rs11991877
rs55747751
rs75664430
rs17677991
rs3767952
rs150150565
rs111950327
rs76065543
rs3891167
rs28502153
rs8102497
rs202034370
rs77231040
rs6669563
rs9419958
rs9600019
rs6584579
rs17445108
rs6776756
rs137901416
rs11426156
rs11117354
rs35446936
rs144204502
rs77732866
rs2967355
rs11699829
rs1291143
rs4731541
rs59409453
rs11557154
rs28363070
rs871134
rs10905255
rs11579626
rs9940099
rs35640778
rs2282764
rs76666449
rs11412296
rs2276182
rs112394943
rs45604339
rs11085072
rs4498805
rs185174247
rs143190905
rs117512405
rs131797
rs16978028
rs6587577
rs6536702
rs117407747
rs76219171
rs80116508
rs6881568
rs28577594
rs13230646
rs7705526
rs1907702
rs611646
rs10845387
rs7790856
rs8105767
rs141214782
rs12932179
rs932002
rs117630647
rs17803849
rs762679
rs139669835
rs79977579
rs376641875
rs11769630
rs1985369
rs7221585
rs80324517
rs12369950
rs66731853
rs10805346
rs117034449
rs2056726
rs111527438
rs4695407
rs142426306
rs12412214
rs939916
rs10112752
rs2977608
rs4530278
rs11584821
rs41269079
rs116863223
rs139795227
rs10840270
rs10768683
rs1957937
rs73581419
rs869785
rs9398196
rs78491606
rs61748181
rs11212631
rs6659669
rs6007020
rs2555104
rs9878436
rs4743037
rs2538745
rs56061761
rs1003322
rs7209057
rs113525195
rs10774624
rs3093888
rs7099229
rs1023767
All − MR Egger
All − Inverse variance weighted
−2
−1
0
1
2
MR effect size for
'telomere length || id:ieu−b−4879' on 'IDP_dMRI_ProbtrackX_L2_cgc_r || id:ub
